# Supplementary material for: ALD Deposited ZnO:Al Films on Mica for Flexible PDLC Devices
Source: Nanomaterials (Basel). 2021 Apr 15;11(4):1011. doi: 10.3390/nano11041011 (PMC8071305; doi:10.3390/nano11041011)
Supplement: Supplementary file 1 [file nanomaterials-11-01011-s001.zip › supplementary/supplementary.pdf]

The Ohmic behavior is supported by measuring the current vs. voltage (*I–V*) characteristics of AZO films on mica shown in Figure S1. By using the formula $R_s = 1/\sigma d$ (where σ is the electrical conductivity of the material and d is the thickness of the electrode) calculated sheet resistance value is also shown in Figure S1.

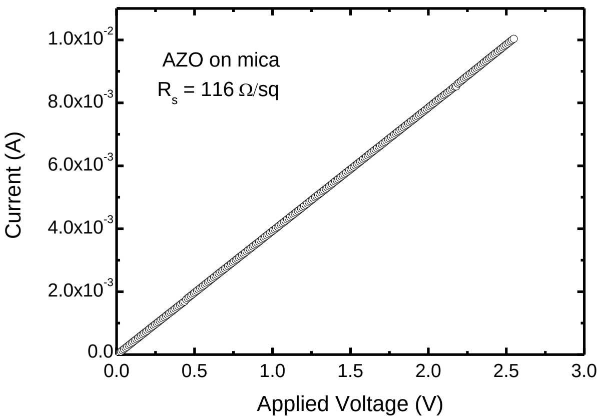

Figure S1. Current-voltage characteristic of AZO thin films deposited on mica substrate.

Photographs of the AZO on mica samples and bending test experimental set-up are shown in Figure S2 (a,b). The bending diameter of 5 × 5 cm size AZO on mica samples was about 3 mm.

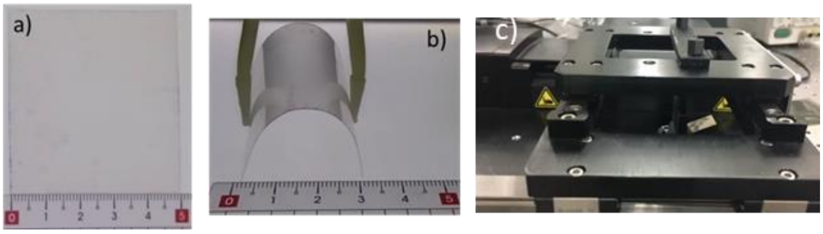

Figure S2. (**a**,**b**) Photographs of the AZO on mica sample and (**c**) bending test experimental set-up.
